# Cu(II), Ni(II), and Zn(II) Complexes of Salan-Type Ligand Containing Ester Groups: Synthesis, Characterization, Electrochemical Properties, and *In Vitro* Biological Activities

**DOI:** 10.1155/2013/439848

**Published:** 2013-07-25

**Authors:** P. Jeslin Kanaga Inba, B. Annaraj, S. Thalamuthu, M. A. Neelakantan

**Affiliations:** Chemistry Research Centre, National Engineering College, K.R. Nagar, Kovilpatti, Thoothukudi District, Tamil Nadu 628 503, India

## Abstract

A salen ligand on reduction and N-alkylation affords a novel [N_2_O_2_] chelating ligand containing ester groups [L = diethyl-2,2′-(propane-1,3-diylbis((2-hydroxy-3-methoxy benzyl)azanediyl))diacetate]. The purity of the ligand was confirmed by NMR and HPLC chromatograms. Its Cu(II), Ni(II), and Zn(II) complexes were synthesized and characterized by a combination of elemental analysis, IR, NMR, UV-Vis, and mass spectral data, and thermogravimetric analysis (TG/DTA). The magnetic moments, UV-Vis, and EPR spectral studies support square planar geometry around the Cu(II) and Ni(II) ions. A tetrahedral geometry is observed in four-coordinate zinc with bulky N-alkylated salan ligand. The redox properties of the copper complex were examined in DMSO by cyclic voltammetry. The voltammograms show quasireversible process. The interaction of metal complexes with CT DNA was investigated by UV-Vis absorption titration, ethidium bromide displacement assay, cyclic voltammetry methods, and agarose gel electrophoresis. The apparent binding constant values suggest moderate intercalative binding modes between the complexes and DNA. The *in vitro* antioxidant and antimicrobial potentials of the synthesized compounds were also determined.

## 1. Introduction

Salen metal complexes are the interest of many workers because of their applications in food industry, in the treatment of cancer [1], as antibactericide agents [2, 3], as antivirus agents [4], as fungicide agents [5], and for other biological properties [6]. The antitumor activity of salen complex arises due to its DNA binding properties. The salen complexes are conformationally flexible and adopt a variety of geometries. Also, salen metal complexes have a unique flat electron-rich aromatic surface that may facilitate their interactions with nucleic acids. Hydroxyl groups in the salen complexes act as a quinone system which would cooperate to facilitate the formation of free radicals responsible for DNA cleavage [7]. The biological properties of salen complexes are enhanced by functionalization with a variety of substituents [8–11]. When salen compounds are reduced at the imine function, the more flexible, reduced salen derivatives (salan) are obtained.

Considerable attention has been devoted to the preparation and structural characterization of metal complexes containing salen-type ligands. However, little attention has been paid to systems in which functionalized salan is used as ligands. In the present investigation N-alkylated salan complexes are used for DNA binding and antimicrobial and antioxidant properties. In continuation of our earlier works on salen-type ligands [12–14], the present investigation reports on the synthesis and spectral characterization of Cu(II), Ni(II), and Zn(II) complexes with N-alkylated salan ligand. The interaction of the metal complexes with calf thymus (CT) DNA was studied by UV-Vis and fluorescence spectroscopy and cyclic voltammetric method. The DNA cleaving nature of the compounds was tested against pUC19 DNA in the absence and presence of hydrogen peroxide. The *in vitro* antimicrobial activity of the compounds was assessed against various microorganisms. The antioxidant activity of the metal complexes was investigated systematically. 

## 2. Experimental

### 2.1. Materials and Methods

All chemicals employed for the synthesis were of analytical reagent grade and of highest purity available. *o-*Vanillin and 1,3-diaminopropane were purchased from Sigma Aldrich and used as received. Solvents used for spectroscopic and electrochemical studies were purified and dried by standard procedures [15]. Metal acetates were purchased from Merck. CT DNA and pUC19 DNA were purchased from GeNei, Bangalore, and used without purification. Tris-(hydroxylmethyl)-aminomethane-HCl (Tris-HCl) and ethidium bromide (EB) were obtained from HiMedia. Tris-HCl-NaCl buffer solution was prepared with double-distilled water. Tetrabutylammonium perchlorate (TBAP) was used as a supporting electrolyte for recording cyclic voltammograms.

### 2.2. Physical Measurements

Elemental analyses were recorded on a Thermo Finnigan Flash EA 1112 elemental analyzer. Molar conductance values of the complexes in DMSO were obtained on a Systronics Model 611 digital conductivity meter. Magnetic susceptibility measurements on powder samples were carried out on a Gouy balance at room temperature using mercuric tetra(thiocyanato)cobaltate (II) as the calibrant. The infrared (IR), ultraviolet-visible (UV-Vis), and emission spectra were recorded on Shimadzu 8400 S, Shimadzu UV-2450, and Shimadzu RF-5301 PC spectrophotometers, respectively. The ^1^H and ^13^C NMR of ligand in CDCl_3_ and zinc complex in DMSO-d_6_ were recorded on Bruker AV 300 MHz spectrophotometer. Electrospray ionization (ESI) mass spectral measurements were recorded on Micromass Quattro II mass spectrometer. Electron paramagnetic resonance (EPR) spectrum of the Cu complex in DMSO solution was recorded on JES-FA200 spectrometer at 300 K and at 77 K using tetracyanoethylene (TCNE, *g* = 2.00277) as the *g* marker. The thermogravimetric analysis/differential thermal analysis (TGA/DTA) was carried out in dynamic nitrogen atmosphere with a heating rate of 20°C/min using NETZSCH STA 449F3 thermal analyzer. Cyclic voltammograms were recorded on a CHI 603 C electrochemical analyzer with a three-electrode compartment.

### 2.3. Synthesis of the Ligand (L)

The synthetic procedure of the ligand was reported in our earlier work [14]. Color: yellow oil. Yield: 60%; Analytical data. Calculated data, for [C_27_H_38_N_2_O_8_] (%): C, 62.53; H, 7.38; N, 5.40. Found (%): C, 62.67; H, 7.25; N, 5.48. IR (cm^−1^): 1273 (phenolic –C–O), 1195 (–C–N), and 1747 ester (–C=O). ^1^H NMR (CDCl_3_): 4.16 (H9, 4H), 3.86 (H5, H11, 10H), 3.35 (H8, 4H), 6.58–6.87 (aromatic-H, 6H), 2.63 (H6, 4H), and 1.77 (H7, 2H) *δ*. *λ*_max⁡_ in CH_3_OH, 267, 333 nm.

### 2.4. Synthesis of the Metal Complexes

**Scheme 1:**
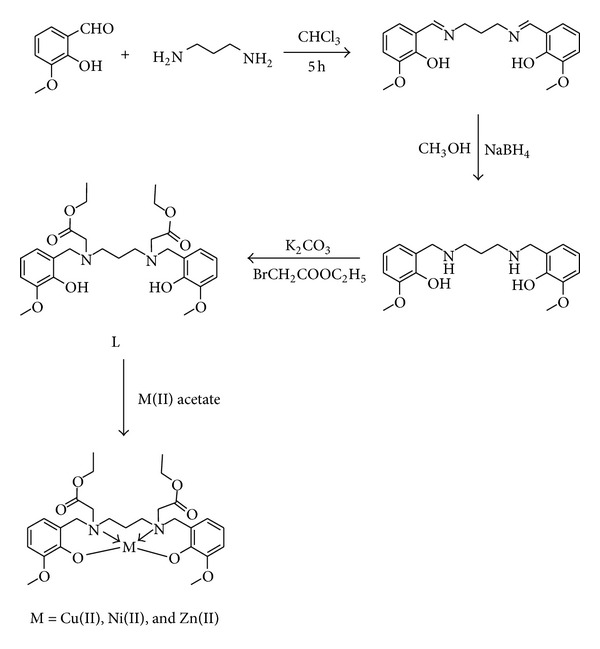
Synthesis route for ligand and metal complexes.

The metal complexes were isolated as follows: metal acetate (2 mmol) dissolved in 10 mL of methanol was added dropwise to a 10 mL methanolic solution of the ligand (2 mmol). The mixture was kept under reflux for 1-2 hours. The solution thus obtained gave the compound on concentration and cooling. All the compounds are soluble in DMSO. The yields were around 70–80%. The synthesis is given in Scheme 1. For the crystallization, the compounds were dissolved in different solvent mixtures and evaporated slowly at room temperature. But our attempts to crystallize the compounds were unsuccessful.

#### 2.4.1. [CuL]·H_2_O

Colour: dark brown; anal. calcd for [Cu(C_27_H_36_N_2_O_8_)]·H_2_O (%): C, 54.22; H, 6.40; N, 4.68; Cu, 10.62. Found (%): C, 54.35; H, 6.45; N, 4.76; Cu, 10.67. IR (cm^−1^): 1265 (phenolic –C–O), 1181 (–C–N), 1749 (–C=O in ester group), 584 (M–N), and 461 (M–O). *m/z*: 580. *λ*_max⁡_ in DMSO 285, 349, and 527 nm. *μ*_eff_: 1.85 BM. Λ_m_: 12.72 mho cm^2 ^mol^−1^.

#### 2.4.2. [NiL]·H_2_O

Colour: red; anal. calcd for [Ni(C_27_H_36_N_2_O_8_)]·H_2_O (%): C, 54.66; H, 6.46; N, 4.72; Ni, 9.89. Found (%): C, 54.71; H, 6.50; N, 4.65; Ni, 9.97. IR (cm^−1^): 1261 (phenolic –C–O), 1187 (–C–N), 1747 (–C=O in ester group), 551 (M–N), and 470 (M–O). ^1^H NMR (DMSO-d_6_): 4.21 (H9, 4H), 3.91 (H11, 6H), 4.37 (H5, 4H), 3.70 (H8, 4H), 6.57–6.94 (aromatic-H, 6H), 3.20 (H6, 4H), and 1.85 (H7, 2H) *δ*. *m/z*: 575. *λ*_max⁡_ in DMSO 280, 345, and 553 nm. Λ_m_: 11.56 mho cm^2 ^mol^−1^.

#### 2.4.3. [ZnL]·H_2_O

Colour: pale yellow; anal. calcd for [Zn(C_27_H_36_N_2_O_8_)]·H_2_O (%): C, 54.05; H, 6.38; N, 4.67; Zn, 10.90. Found (%): C, 54.12; H, 6.46; N, 4.81; Zn, 10.97. IR (cm^−1^): 1267 (phenolic –C–O), 1183 (–C–N), 1749 (–C=O in ester group), 547 (M–N), and 451 (M–O). ^1^H NMR (DMSO-d_6_): 4.19 (H9, 4H), 3.92 (H11, 6H), 4.35 (H5, 4H), 3.72 (H8, 4H), 6.55–6.91 (aromatic-H, 6H), 3.16 (H6, 4H), and 1.79 (H7, 2H) *δ*.* m/z*: 582. *λ*_max⁡_ in DMSO 282 and 350 nm. Λ_m_: 13.28 mho cm^2 ^mol^−1^. 

### 2.5. DNA Binding Experiments

#### 2.5.1. UV-Vis Spectroscopic Studies

The DNA binding experiments were performed at room temperature. A solution of CT DNA in the buffer (5 mM Tris-HCl and 50 mM NaCl) gave a ratio of UV absorbance at 260 and 280 nm of about 1.8–1.9 : 1, indicating that the CT DNA was sufficiently free from protein [16]. The concentration of DNA was measured using its extinction coefficient at 260 nm (6600 mol L^−1 ^cm^−1^) [17]. Concentrated stock solutions of the compounds in DMSO were prepared and diluted suitably with the buffer to the required concentrations for all the experiments. The absorption titrations of the compounds in buffer were performed using a fixed concentration (10 *μ*M) to which increments of the DNA stock solution were added (*R* = [DNA]/[complex] = 0, 2, 4, 6, 8, and 10). Compound DNA solutions were allowed to incubate for 30 min before the spectra were recorded. From the absorption data, the intrinsic binding constant, *K*_*b*_, was determined using the following [18]:

(1) $$\begin{array}{c} \frac{\left[\text{DNA}\right]}{\left(\varepsilon_{a}-\varepsilon_{f}\right)}=\frac{\left[\text{DNA}\right]}{\left(\varepsilon_{b}-\varepsilon_{f}\right)}+\frac{1}{K_{b}\left(\varepsilon_{b}-\varepsilon_{f}\right)}, \end{array}$$

where *ε*_*a*_, *ε*_*f*_, and *ε*_*b*_ are the apparent, free, and bound compound extinction coefficients, respectively. In the plots of [DNA]/(*ε*_*a*_ − *ε*_*f*_) versus [DNA], *K*_*b*_ is given by the ratio of slope to the intercept. 

#### 2.5.2. Fluorescence Studies

The interaction of the synthesized compounds with DNA was further studied by ethidium bromide (EB) displacement method. The excitation wavelength was fixed at 530 nm, and the emission range was adjusted before measurements. The changes in the fluorescence intensities at 595 nm of EB-bound CT DNA in Tris-HCl buffer (pH 7.2) were measured with respect to different concentrations of the compounds (0–120 *μ*M). The magnitude of the binding strength of the compounds with CT DNA can be calculated using linear Stern-Volmer equation [19]:

(2) $$\begin{array}{c} \frac{I_{0}}{I}=1+K_{\text{sv}}\left[Q\right], \end{array}$$

where *I*_0_ and *I* are the fluorescence intensities of EB-DNA in the absence and presence of quencher, respectively, *Q* is the concentration of metal complex, and *K*_sv_ is linear Stern-Volmer quenching constant. The relative binding tendency of the complex to CT DNA was determined by the comparison of the slope of the line in the fluorescence intensity versus complex concentration plot. The apparent binding constant (*K*_app_) was calculated using the equation *K*_app_ = *K*_EB_[EB]/[complex], where [complex] is the concentration of the complex at which there is 50% reduction in the fluorescence intensity of EB, *K*_EB_ = 1.0 × 10^7^ M^−1^, and [EB] = 5 *μ*M [20].

#### 2.5.3. Electrochemical Studies

The cyclic voltammetric studies of the copper complex were performed with a three-electrode system of glassy carbon as working electrode, Pt wire as auxiliary electrode, and Ag/AgCl as reference electrode. The supporting electrolyte is 0.05 M TBAP in DMSO solution. The cell was maintained oxygen-free by passing dry nitrogen through the solution. The interaction of the copper complex with CT DNA has been investigated by monitoring the changes observed in the cyclic voltammogram of CuL in buffer (5 mM Tris-HCl/50 mM NaCl) with increasing amount of DNA. 

### 2.6. DNA Cleavage Experiment

The DNA cleavage experiment was conducted by gel electrophoresis on pUC19 DNA. The reaction mixture was prepared as follows: 1 *μ*L of pUC19 DNA, 5 *μ*L of the compound in DMSO, and 1 *μ*L of H_2_O_2_ followed by dilution with buffer (50 mM Tris-HCl and 50 mM NaCl) to a total volume of 25 *μ*L. The reaction mixture was incubated at 37°C for 1 h. The 1% agarose gel was prepared and stained using ethidium bromide. The samples were then loaded on gel after mixing with 3 *μ*L of loading dye (0.25% bromophenol and 40% sucrose). The gel was electrophoresed at 100 V using Tris-boric acid-EDTA buffer (pH = 8.0) until the bromophenol blue reached one-third of the gel. The bands were visualized and photographed under a UV transilluminator. The experiment was also carried out in the absence of H_2_O_2_.

### 2.7. Antioxidant Property

2,2′-diphenyl-1-picrylhydrazyl (DPPH•) scavenging capacity (antioxidant activity) was measured according to the following procedure [21, 22]. The concentration of DPPH• used for antioxidant activity was 50 *μ*M. Different concentration of the ligand and metal complexes in methanol was added to DPPH• in methanol solution and kept at room temperature for 30 min in dark. The reduction of the DPPH• was monitored by observing the decrease in absorbance at 517 nm using UV-Vis spectrophotometer. The radical scavenging capacity of the antioxidant was expressed in terms of % inhibition and IC_50_. The capability to scavenge the DPPH• was calculated using the following [23]:

(3) $$\begin{array}{c} \%\;\text{Inhibition}=\left(\frac{A_{0}\;-\;A_{\text{sample}}}{A_{0}}\right)\times 100, \end{array}$$

where *A*_0_ is the absorbance of DPPH• in methanol solution without an antioxidant and *A*_sample_ is the absorbance of DPPH• in the presence of an antioxidant. The IC_50_ value is the concentration of the antioxidant required to scavenge 50% DPPH• and is calculated from the inhibition curve. 

### 2.8. Antimicrobial Activity

All the synthesized compounds were screened for their antibacterial activity against gram-positive bacteria: *Streptococcus pyogenes* and *Staphylococcus aureus*, and gram-negative bacteria: *Escherichia coli*,* Klebsiella mobilis, Aeromonas aquariorum*, and *Serratia marcescens*, by well diffusion method [24]. Standard antibiotics, ampicillin and amoxicillin, were used as controls. Stock solutions of tested compounds were prepared in DMSO to a final concentration of 10 mg mL^−1^. 20 mL of sterilized agar media was poured into each presterilized Petri dish and allowed to solidify by placing it in an incubator at 37°C for an hour. 24 h culture suspension was poured and neatly swabbed with the presterilized cotton swabs. Then holes of 5 mm diameter were punched carefully using a sterile cork borer, and these wells were completely filled with the prepared L or the metal complex solutions (50 *μ*L). These dishes were transferred to an incubator maintained at 37°C for 24 h. During this period, the test solution diffused and the growth of the inoculated microorganism was affected. The inhibition zone was developed and measured at the end of the incubation period. Experiments were performed in triplicate, and standard deviation was calculated. 

## 3. Results and Discussion

The ligand was synthesized by three steps. In the first step Schiff's base was obtained by the condensation of o-vanillin with 1,3-diaminopropane. The Schiff base was reduced using sodium borohydride in the second step. Finally ester compound is obtained by N-alkylation reaction using 2-bromoethylacetate in the presence of potassium carbonate. The ester compound obtained as yellow oily substance on complexation with metal ions forms powdered metal complexes. 

### 3.1. Molar Conductance

The molar conductance of the synthesized metal complexes was measured in DMSO at 10^−3 ^M solution. The values were found to be in the range of 12.72–16.56 mho cm^2 ^mol^−1^ suggesting the nonelectrolytic nature of the complexes.

### 3.2. IR Spectra

The ligand (L) has two characteristically strong bands (1747 and 1205 cm^−1^) arising from C=O and C(O)–O stretching vibrations of ester groups (Figure S1) (see Supplementary Material available online at http://dx.doi.org/10.1155/2013/439848). These vibrations are unchanged in the spectra of the complexes indicating that the ester group of L is not involved in complexation with metal ion. The ligand shows two bands at 1379 and 1280 cm^−1^ corresponding to O–H bending and C–O stretching vibrations of phenolic OH group. The disappearance of O–H bending and higher shifting of C–O stretching vibrations are observed in the spectra of metal complexes suggesting that the phenolic OH group of L is involved in coordination with metal ion after deprotonation. The band at 1193 cm^−1^ assigned for C–N stretching in the free ligand is shifted to lower wave number in complexes. This suggests that the tertiary nitrogen atom of the ligand is involved in coordination with the metal ions. In all the complexes, a broad band that appears in the region 3400–3500 cm^−1^ shows the existence of uncoordinated water molecule. New bands which are not present in the ligand appeared in the ranges 505–584 cm^−1^ and 450–470 cm^−1^ in the complexes attributed to *ν*_(*M*–*N*)_ and *ν*_(*M*–*O*)_ vibrations, respectively. From the spectral data, the ligand coordinates to the metal ion through phenolic –O and tertiary –N atoms. 

### 3.3. NMR Spectra

Formation of nickel and zinc complex is confirmed by comparing the ^1^H NMR of ligand and its metal complex (Table S1). The N-methylene protons (H8) of ester part of L give the singlet at 3.35 *δ*. The methylene protons (H6) *α* to amino part of L show the signal at 2.63 *δ*. The sharp singlet at 3.86 *δ* corresponds to methylene protons (H5) *α* to phenyl ring (Figure S2). These methylene proton signals undergo higher deshielding up to 0.1 to 0.5 *δ*. This demonstrates that the tertiary amine nitrogen is involved in coordination with metal ion. The ligand shows that multiple signals at 4.16 and 1.43 *δ* are assignable to methylene and methyl protons of ester groups. These signals are not altered in the metal complex (Table S1). This suggests that the ester group of L is free from coordination with the metal ions. The aromatic protons of L show multiple signals in the region 6.58–6.87 *δ*. The sharp singlet at 3.86 *δ* corresponds to methoxy protons. These protons undergo smaller deshielding up to 0.06 *δ*.

**Figure 1:**
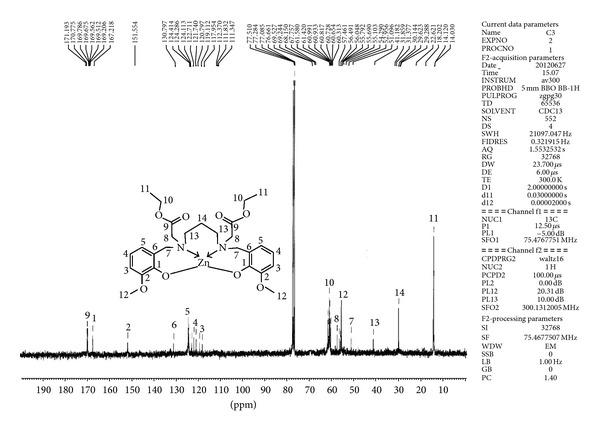
^13^C NMR spectrum of ZnL recorded in DMSO-d_6_.

The ^13^C NMR spectral data of L is compared with its ZnL complex (Figure S3 and Figure 1). The ^13^C NMR signal for ester group of L (C-9) is not altered in the zinc complex (Table S2). This suggests that the ester group of L is free from coordination with the metal ion. The signals for carbon atoms adjacent to nitrogen (C-7 and C-13) are observed at 54.20 and 45.37 *δ*, respectively. These signals are shifted to lower value in the metal complexes. Similarly, the carbon atom adjacent to phenolic oxygen (C-1) of L is shifted to higher value in the ZnL complex. The shifts in the positions of the carbon atoms adjacent to nitrogen and phenolic oxygen clearly demonstrate the bonding of the two nitrogen atoms of tertiary amine and two oxygen atoms of phenol to the Zn(II) ion forming tetrahedral geometry.

### 3.4. Electronic Absorption Spectra

The UV-Vis absorption spectra of L and its metal complexes in DMSO were recorded at room temperature (Figure S4). The absorption spectrum of L shows bands at 267 and 335 nm, which are due to *π* → π* transitions of phenyl ring and H-bonding induced changes of OH proton-donor aromatic molecules and amine NH (intraligand charge transfer band), respectively. The spectrum of CuL (Figure S5) displaying the band at 527 nm is assigned to ^2^ *B*_1*g*_ →^2^ *A*_1*g*_ transition confirming the square planar geometry of the CuL (Table 1). The magnetic moment value for CuL (1.85 BM) is consistent with the square planar Cu(II) system. The NiL complex showed absorption at 553 nm ascribed to d-d transition (^1^ *A*_1*g*_ → ^1^ *A*_2*g*_) which supports the square planar geometry around Ni(II) ion [25].

### 3.5. Mass Spectra

ESI mass spectra of all the metal complexes support the proposed structure of the complexes. The copper complex shows main peak at *m/z* 580 corresponding to the molecular weight of the complex (Figure S6). The fragmentation peaks of copper complex are observed at *m/z* 379 and 491. The molecular ion and fragmentation peaks have half intensity peaks due to isotopic distributions of copper (^63^Cu and ^65^Cu) [26, 27]. The spectral result shows that metal complexes are monomeric in nature and the metal to ligand ratio is 1 : 1. Nickel and zinc complexes are the same as copper, supported by analytical and spectral analysis.

### 3.6. EPR Spectra

The X-band EPR spectrum of the copper complex was recorded at 300 K and at 77 K using TCNE as the *g* marker (Figure S7). The absence of a half-field signal at 1600 G due to the *m*_*s*_ = ±2 transitions ruling out any Cu-Cu interaction suggests the monomeric nature of the CuL complex. The observed *g* values are *g*_II_(2.29) > *g*_⊥_(2.11) > *g*_*e*_ (2.0027), suggesting the unpaired electron is in the *d*_x2−y2  _ orbital (Table 2). The *g*_II_/*A*_II_ value calculated for CuL (138 cm) lies between 90 and 140 cm indicating a square planar structure around the Cu (II) ion [28–30]. The *g*_II_ value of 2.29 for the CuL complex indicates the covalent nature of the metal-ligand bond. The *g* values are related to exchange interaction coupling constant (*G*) by the expression *G* = (*g*_II_ − 2.0027)/(*g*_⊥_ − 2.0027). If *G* < 4, the ligand forming the copper complex is regarded as a strong-field ligand. For the present square planar complex, *G* = 2.67 indicates that the ligand is strong field and the metal-ligand bonding in the complex is covalent [31].

The bonding parameters *α* ^2^, *β* ^2^, and *γ* ^2^ which may be regarded as covalency of the in-plane *σ* bond, in-plane *π* bond, and out-of-plane *π* bond, respectively, were evaluated from the following expressions [32]:

(4) $$\begin{array}{c} \alpha^{2}=-\frac{A_{II}}{0.036}+\left(g_{\text{II}}-2.0027\right)+\frac{3}{7}\left(g_{⊥}-2.0027\right)+0.04, \end{array}$$

(5) $$\begin{array}{c} \beta^{2}\;=\;\left(g_{\text{II}}-2.0027\right)\frac{E}{-8\lambda \alpha^{2}}, \end{array}$$

(6) $$\begin{array}{c} \gamma^{2}\;=\;\left(g_{⊥}-2.0027\right)\frac{E}{-2\lambda \alpha^{2}}, \end{array}$$

where *λ* = −828 cm^−1^ for Cu(II) d^9^ system and *E* is the electronic transition energy. The *α* ^2^ value of 0.83 for CuL demonstrates that the complex has covalent character in the ligand environment. The observed *β* ^2^ and *γ* ^2^ values indicate that there is an interaction in the in-plane *π* bonding between the metal ion and ligand. This is also confirmed by orbital reduction factors, *K*_II_ and *K*_⊥_: *K*_II_ = *α* ^2^ *β* ^2^ and *K*_⊥_ = *α* ^2^ *γ* ^2^. In the present investigation the trend *K*_II_ < *K*_⊥_ for the copper complex implies a considerable in-plane *π* bonding is between the metal ion and the ligand [33]. 

### 3.7. Thermal Studies

The metal complexes show gradual loss in weight due to the decomposition with increasing temperature (Figure S8). The thermogram shows four decomposition steps within the temperature range of 25–1000°C. In the first step upto 100°C, the mass loss (3–4.2%) corresponds to loss of lattice water molecule. In the second step (100–275°C), the mass loss of 22–25% corresponds to removal of the ester and methoxy groups with evolution of CO_2_ gas. The third step of decomposition is noticed in the temperature range 275–450°C with loss of 28–30% due to the removal of the amino part of the ligand in the complexes. The fourth stage of decomposition occurs in the range 450–1000°C, which corresponds to the removal of the remaining part of the ligand leaving metal oxide as a residue.

### 3.8. DNA Binding Experiments

#### 3.8.1. Absorption Spectroscopic Studies

**Figure 2:**
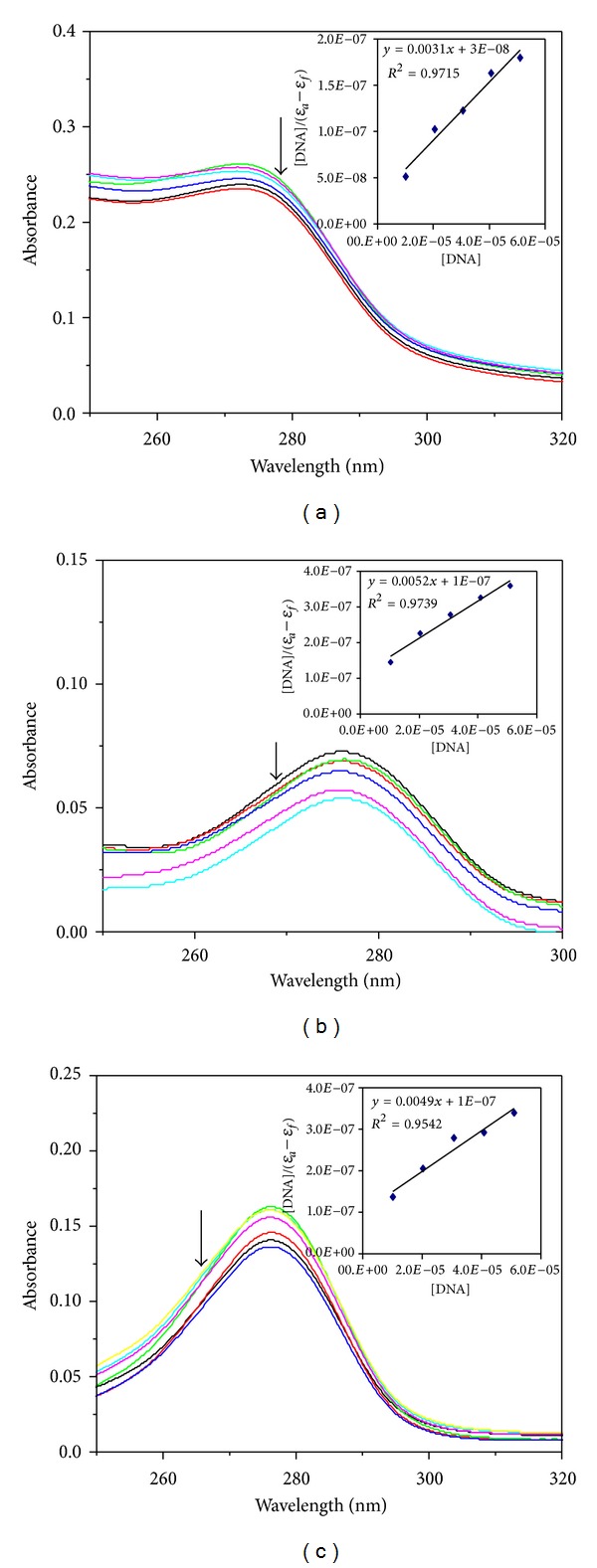
UV-Vis absorption spectra of (a) CuL, (b) NiL, and (c) ZnL (10 *µ*M) in the absence and presence of increasing amounts of CT DNA; [DNA] = 0–60 *µ*M. The arrow indicates the absorbance change upon increasing DNA concentration. The inset is a plot of [DNA]/(*ε*_*a*_ − *ε*_*f*_) versus [DNA].

All the metal complexes show intraligand (*π* → π*) transition in the region 270–280 nm. On addition of DNA, this band of the complexes was affected resulting in the tendency of hypochromism lying in the range 20–32% and a slight bathochromic shift in the range of 1.6-1.7 nm (Figure 2). These phenomena indicate that the complexes probably interact with CT DNA by intercalation binding mode. The extent of hypochromism is commonly consistent with the strength of intercalative interaction [34]. In order to study the binding ability of the compounds with CT DNA, the binding constant, *K*_*b*_, was determined. The *K*_*b*_ values of the ligand (1.23 × 10^5^ M^−1^) and its copper complex (1.00 × 10^5^ M^−1^) are comparable. But the *K*_*b*_ values are found to be lower than those reported for typical intercalators (for ethidium bromide and [Ru(Phen)_2_(dppz)]^2+^; the binding constants have been found to be of the order 1.4 × 10^6^ and >10^6^ M^−1^) [35]. The *K*_*b*_ values indicate that the binding strength of the N-alkylated salan ligand (L) and CuL with DNA is stronger than that of salen, salan [14], NiL, and ZnL (Table 1).

#### 3.8.2. Fluorescence Spectral Studies

**Figure 3:**
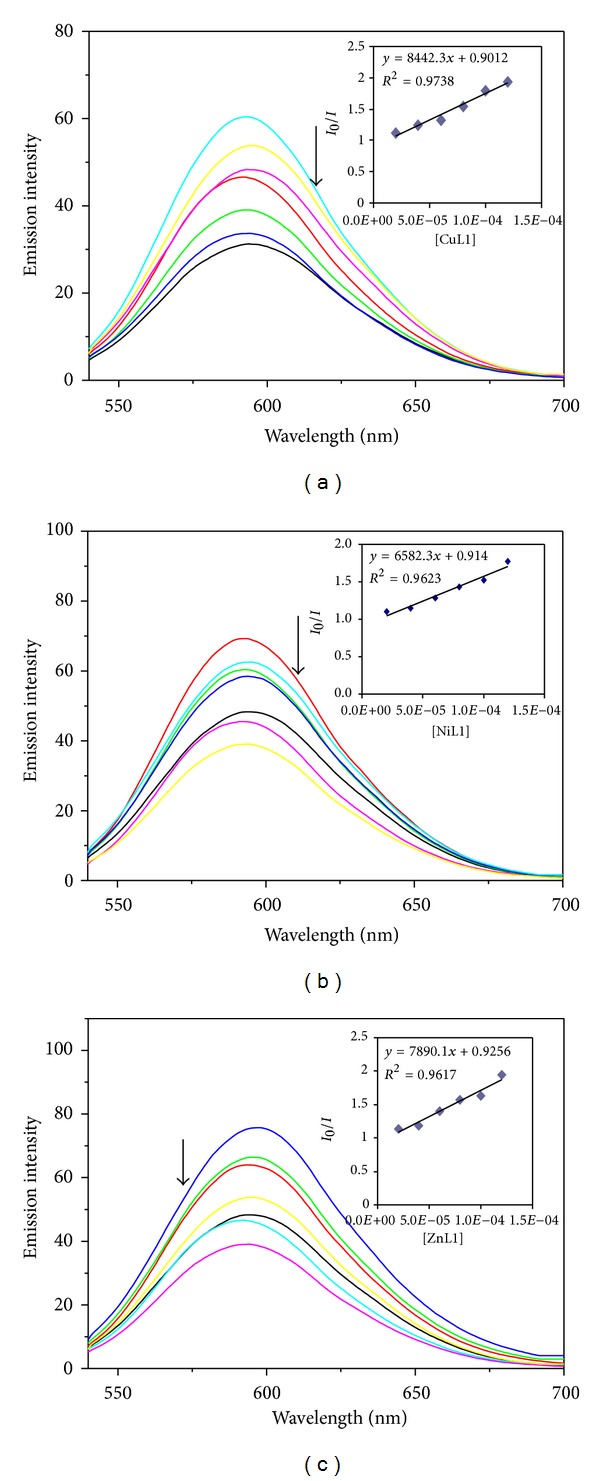
Emission spectra of ethidium-bromide-bound DNA in the presence of (a) CuL, (b) NiL, and (c) ZnL. The arrow shows the intensity change upon increasing complex concentrations. Inset shows the plot of *I*_0_/*I* Vs [complex].

The EB fluorescence displacement experiment has been widely used to investigate the interaction of metal complexes with DNA. The EB shows weak fluorescence in buffer solution. The fluorescence intensity of EB in presence of DNA can be greatly enhanced due to intercalation with DNA [36]. On addition of metal complexes (0–120 *μ*M) to DNA-EB mixture, the metal complex competes with EB to bind with DNA. This leads to a decrease in the binding sites of DNA available for EB, and hence quenching of fluorescence intensity of EB-DNA mixture occurs (Figure 3). The quenching plot illustrates that the quenching of ethidium bromide bound to DNA by metal complexes is in agreement with the linear Stern-Volmer equation. The value of *K*_sv_ for CuL, NiL, and ZnL is found to be 8.44 × 10^3^, 6.58 × 10^3^, and 7.89 × 10^3^, respectively. The apparent binding constant (*K*_app_) values obtained for the CuL, NiL, and ZnL compounds are found to be 6.25 × 10^5^, 5 × 10^5^, and 4 × 10^5^, respectively (Table 1). Furthermore, the quenching constants and binding constants calculated for the complexes suggest that the interaction of all the compounds with DNA occurs through intercalation. The DNA binding abilities of the complexes follow the order Cu(II) > Ni(II) > Zn(II), which is in conformity with the trend in DNA binding affinities obtained from absorption spectral studies.

**Figure 4:**
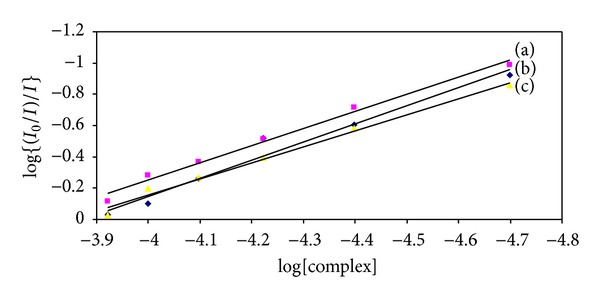
Scatchard plot of fluorescence titration of (a) CuL, (b) NiL, and (c) ZnL with CT DNA.

*Binding Analysis.* The equilibrium binding constant and the number of binding sites can be analyzed according to the Scatchard equation [37, 38]:

(7) $$\begin{array}{c} \log\frac{I_{0}-I}{I}=\log\;K_{\text{bin}}+n\;\log\left[Q\right], \end{array}$$

where *K*_bin_ is the binding constant of complex with DNA and *n* is the number of binding sites. From the plot of log (*I*_0_ − *I*)/*I* versus log⁡[*Q*], the number of binding sites and binding constant have been obtained (Figure 4). The value of *n* is around one for all the compounds indicating the existence of only one independent class of binding sites for the metal complexes on DNA (Table 1). The values of *K*_sv_ and *K*_bin_ suggest that the complexes interact strongly with DNA.

#### 3.8.3. Electrochemical Studies

**Figure 5:**
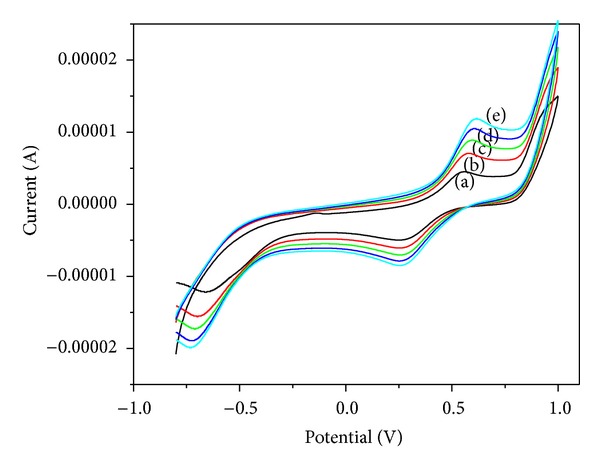
Cyclic voltammogram of CuL at different scan rates: (a) 25 mVs^−1^, (b) 50 mVs^−1^, (c) 75 mVs^−1^, (d) 100 mVs^−1^, and (e) 125 mVs^−1^.

The redox behaviour of the Cu(II) complex in DMSO was examined by means of cyclic voltammetry (potential range −1 V to +1 V) with different scan rates from 25 to 125 mVs^−1^ (Figure 5). The cyclic voltammogram shows a well-defined quasireversible peak for the redox couple Cu(III)/Cu(II) [Ep_a_ = 0.604 V and Ep_c_ = 0.260 V]. In the negative potential range copper shows irreversible cathodic peak at −0.675 V [Cu(II)/Cu(I)] with the scan rate of 100 mVs^−1^ (Table S3). The limiting peak to peak separation (ΔEp) for Cu(III)/Cu(II) process is greater than 59 mV which revealed that the couple is quasireversible. The ratio of anodic to cathodic peak current value is 1.1 demonstrating the simple one-electron process. Further, the anodic peak is shifted towards positive potential value, and the cathodic peak is shifted towards negative potential with a function of scan rate 25 to 125 mVs^−1^ which supports quasireversible process. 

The cyclic voltammetric technique provides information about interaction between the metal complexes and DNA. DNA is denatured in DMSO medium, so we recorded the CV of copper complex in Tris-HCl buffer containing 10% DMSO. In Buffer medium, the copper complex shows a Cu(II)/Cu(I) couple with Ep_c_ at −0.438 V and Ep_a_ at 0.137 V (Figure S9). The separations of anodic and cathodic peaks (ΔEp) are found to be 0.575 V indicating quasireversible one-electron redox process (Table S4). In the presence of CT DNA with *R* = 10 (*R* = [DNA]/[complex]) both the anodic and cathodic peak currents are decreased with shifting of potential values indicating that there exist interactions between copper complex and CT DNA. The drop of the voltammetric current in the presence of CT DNA is due to slow diffusion of the copper complex bound to CT DNA. The formal potential, *E*_1/2_, taken as the average of Ep_*c*_ and Ep_*a*_ shifts slightly towards the positive side on binding to DNA which suggests that copper complex binds intercalatively to CT DNA [39]. 

### 3.9. DNA Cleavage Activity

The cleavage of pUC19 DNA induced by the metal complexes in the presence of H_2_O_2_ is shown in Figure S10. In the absence of the complex (Lane 1), DNA remains in the supercoiled form. Incubation of DNA with copper complex (Lane 2) leads to its conversion to form II and form III. This indicates that copper complex has the ability to cleave pUC19 DNA in presence of oxidant H_2_O_2_. The probable reason may be the oxidation of deoxyribose moiety by hydroxyl free radicals followed by the hydrolytic cleavage of the sugar phosphate backbone. The cleavage efficiency was measured in terms of the ability of the complex to convert the supercoiled form to open circular form. The ability of nickel and zinc complexes (lane 3 and lane 4) in the interconversion of supercoiled form to open circular form (form I to form II) is less when compared to the copper complex. In the absence of H_2_O_2_, the synthesized complexes did not show any effect towards the cleavage of DNA. 

### 3.10. Antioxidant Property

**Figure 6:**
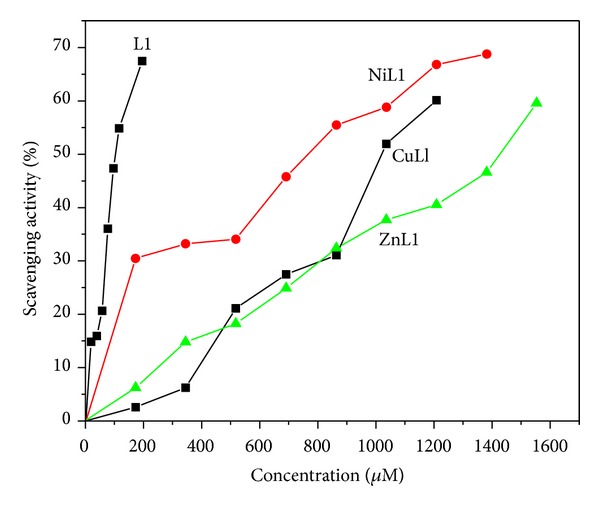
Scavenging activity of the ligand (L) and its Cu(II), Ni(II), and Zn(II) complexes at various concentrations.

The antioxidant activity of the ligand and the metal complexes was measured in terms of their hydrogen donating or radical scavenging capability by DPPH assay method. Upon addition of metal complexes, the reduction of DPPH radical is monitored by the decrease of the absorbance of its radical at 517 nm (Figure 6). The absorbance decreases as a result of color changes from purple to yellow as the radical is scavenged by antioxidants. The 50% inhibitory concentration (IC_50_) values of L, CuL, NiL, and ZnL are 103, 1019, 771, and 1429 *μ*M, respectively. The higher free radical scavenging activity of the ligand may be due to the presence of free phenolic –OH groups. The IC_50_ values of synthesized compounds are much higher than the positive control like ascorbic acid, 11.55 *μ*M [40].

### 3.11. Antibacterial Activity

The *in vitro* antibacterial activities of the synthesized compounds were tested against six human pathogenic microorganisms (gram-positive bacteria:* Streptococcus pyogenes* and *Staphylococcus aureus*; gram-negative bacteria: *Escherichia Coli*,* Klebsiella mobilis, Aeromonas aquariorum*, and *Serratia marcescens*) by well diffusion method using ampicillin and amoxicillin as standards. The susceptibility of the strains of bacteria towards the present compounds was judged by measuring the size of inhibition diameter (Figure S11). The comparison of the antimicrobial activity of the synthesized compounds and the known antibiotics showed that the metal complexes were more effective than the ligand or metal salts but less active than the controls against all the bacteria tested. The bulky N-alkylated ligand on chelation to the metal cation reduces the polarity of the metal ion due to the ligand orbital overlap with the metal orbitals, resulting in a delocalization of positive charge. This increases the lipophilic character of the metal chelate and favors its permeation through the lipoid layer of the bacterial membranes. Cu(II) complex has higher antibacterial activity against *Streptococcus pyogenes *and* Escherichia Coli* than the other metal complexes. Zn(II) complex has higher activity against* Klebsiella mobilis, Aeromonas aquariorum.* Ni(II) complex is found to have moderate activity towards the bacteria tested. 

## 4. Conclusion

Salan-type ligand containing ester groups was synthesized. The N-alkylated salan was used to prepare Cu(II), Ni(II), and Zn(II) complexes. The synthesized compounds were characterized by spectral and analytical techniques. Spectral studies reveal that the ligand coordinates to the metal ion through the phenolic –O and tertiary –N atoms. The presence of ester groups in tertiary –N leads to distortion from the regular square planar geometry of the complexes. The cyclic voltammogram of copper complex reveals that the complex exhibits well-defined quasireversible Cu(III)/Cu(II) couple along with Cu(II) irreversible process. The synthesized compounds bind to CT DNA through intercalation mode. Copper complex has been found to promote cleavage of pUC19 DNA from the super coiled form to nicked form in presence of H_2_O_2_. The metal complexes show higher bacterial activity than the ligand. The ligand shows more effective free radical scavenger activity than the metal complexes.

## Supplementary Material

Analytical and spectral data relevant to this article are given in the supplementary materials as Figures S1-S11 (IR, UV-Vis., ^1^H & ^13^C-NMR, ESI-Mass, EPR, TGA, CV spectra, DNA cleavage Pattern, antimicrobial activity) and Tables S1-S4 (NMR and CV data).

## Figures and Tables

**Table 1:** Absorption and emission spectral properties of metal (II) complexes with CT DNA.

Compound	*λ*_max⁡_ (nm)	Band assignments	*λ*_max⁡_ (nm)		Δ*λ* (nm)	^a^H%	*K*_*b*_ × 10^4^ (M^−1^)	*K*_app_ × 10^5^ (M^−1^)	*K*_sv_ × 10^3^ (M^−1^)	*K*_bin_ × 10^4^ (M^−1^)	*n*
			Free	Bound							
CuL	285, 349, 527	*π* → π*, ^2^ *B*_1*g*_ → ^2^ *A*_1*g*_	271.5	273.2	1.7	32	10	6.25	8.44	3.08	1.159
NiL	280, 345, 553	*π* → π*, ^1^ *A*_1*g*_ → ^1^ *A*_2*g*_	278.1	279.7	1.6	21	5	5	6.58	1.43	1.092
ZnL	282, 350	*π* → π*	274.8	276.4	1.6	20	4	4.16	7.89	0.87	1.024

**Table 2:** Spin Hamiltonian parameters of Cu(II) complex in DMSO at 300 K and 77 K.

Compound	Hyperfine constant 10^−4^ (cm^−1^)												
	*A*_ll_	*A*_⊥_	*A*_av_	*g*_II_	*g*_⊥_	*g*_av_	*g*_ll_/*A*_ll_	K_II_	*K*_⊥_	*α* ^2^	*β* ^2^	*γ* ^2^	*G*
CuL	165	135	145	2.29	2.11	2.17	138	0.82	1.22	0.83	0.99	1.48	2.67
