# Supplementary material for: Cu(II), Ni(II), and Zn(II) Complexes of Salan-Type Ligand Containing Ester Groups: Synthesis, Characterization, Electrochemical Properties, and In Vitro Biological Activities
Source: Bioinorg Chem Appl. 2013 Jul 25;2013:439848. doi: 10.1155/2013/439848 (PMC3741936; doi:10.1155/2013/439848)
Supplement: Supplementary file 1 — Analytical and spectral data relevant to this article are given in the supplementary materials as Figures S1-S11 (IR, UV-Vis., 1H & 13C-NMR, ESI-Mass, EPR, TGA, CV spectra, DNA cleavage Pattern, antimicrobial activity) and Tables S1-S4 (NMR and CV data). [file 439848.f1.doc]

**Supporting Information**

**Cu(II), Ni(II) and Zn(II) complexes of salen type ligand containing ester groups: Synthesis, characterization, electrochemical properties and in vitro biological activities**

**P. Jeslin Kanaga Inba, B. Annaraj, S.Thalamuthu, M.A. Neelakantan***

*Chemistry Research Centre, National Engineering College, Kovilpatti - 628 503, Tamil Nadu, India.*

^∗^Corresponding author: Tel.: +919442505839; fax: +9104632232749.

E-mail address: [drmaneelakantan@gmail.com](mailto:drmaneelakantan@gmail.com), [maneels@rediffmail.com](mailto:maneels@rediffmail.com) (M.A. Neelakantan).


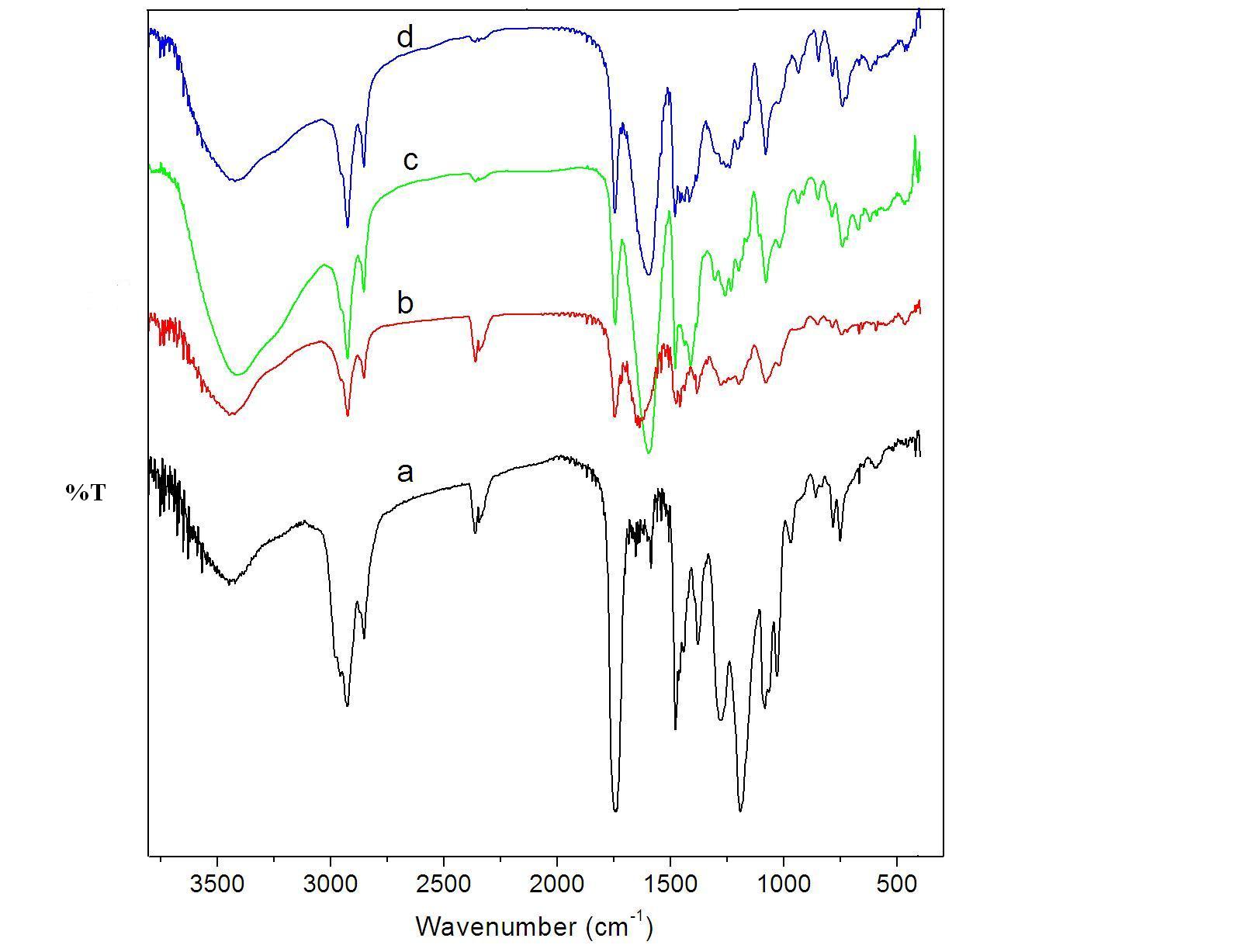


**FIGURE S1:** IR spectra of a) L b) CuL c) NiL and d) ZnL.


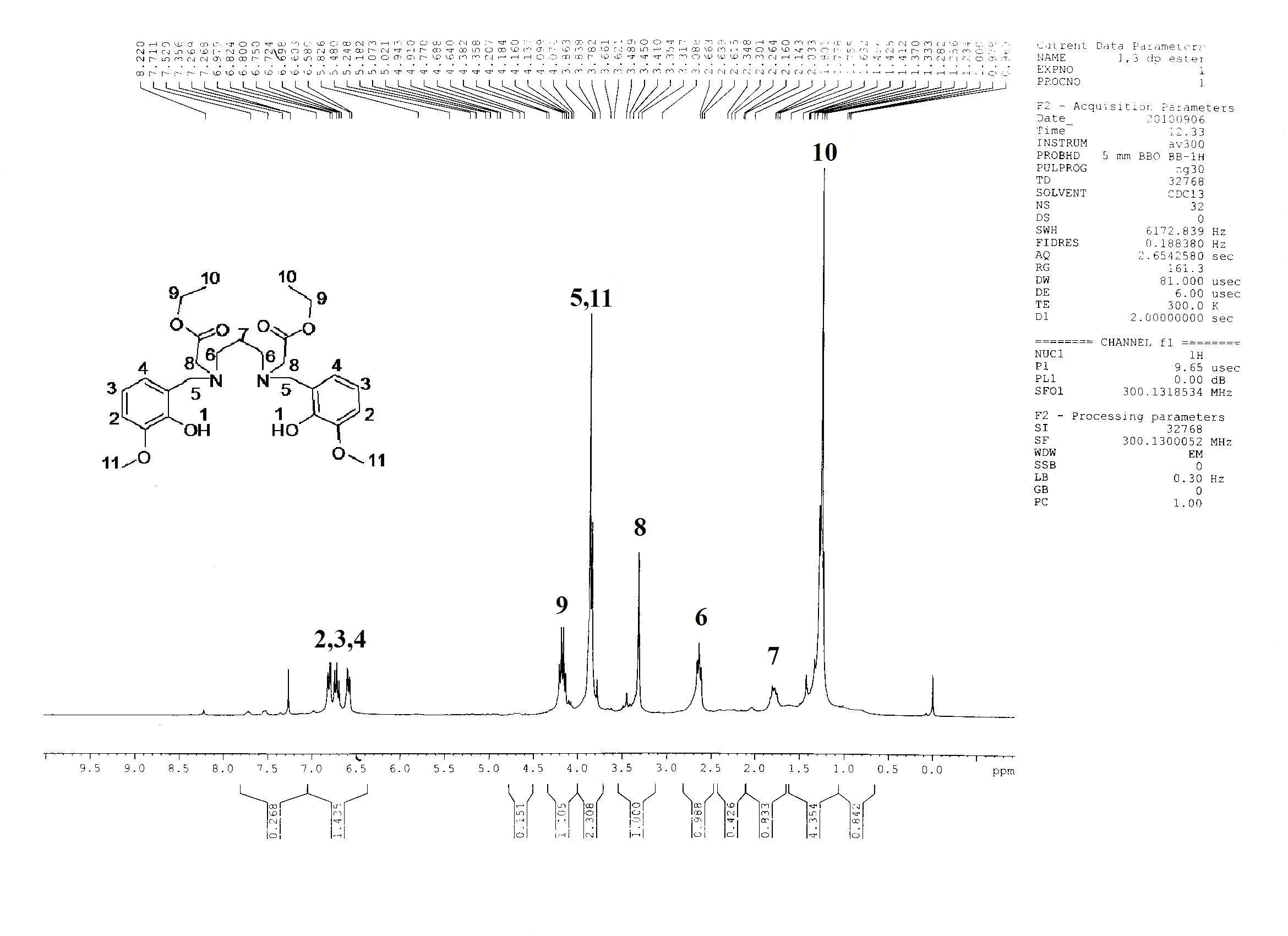


**FIGURE S2:** ^1^H NMR spectrum of **L** in CDCl_3_.

**
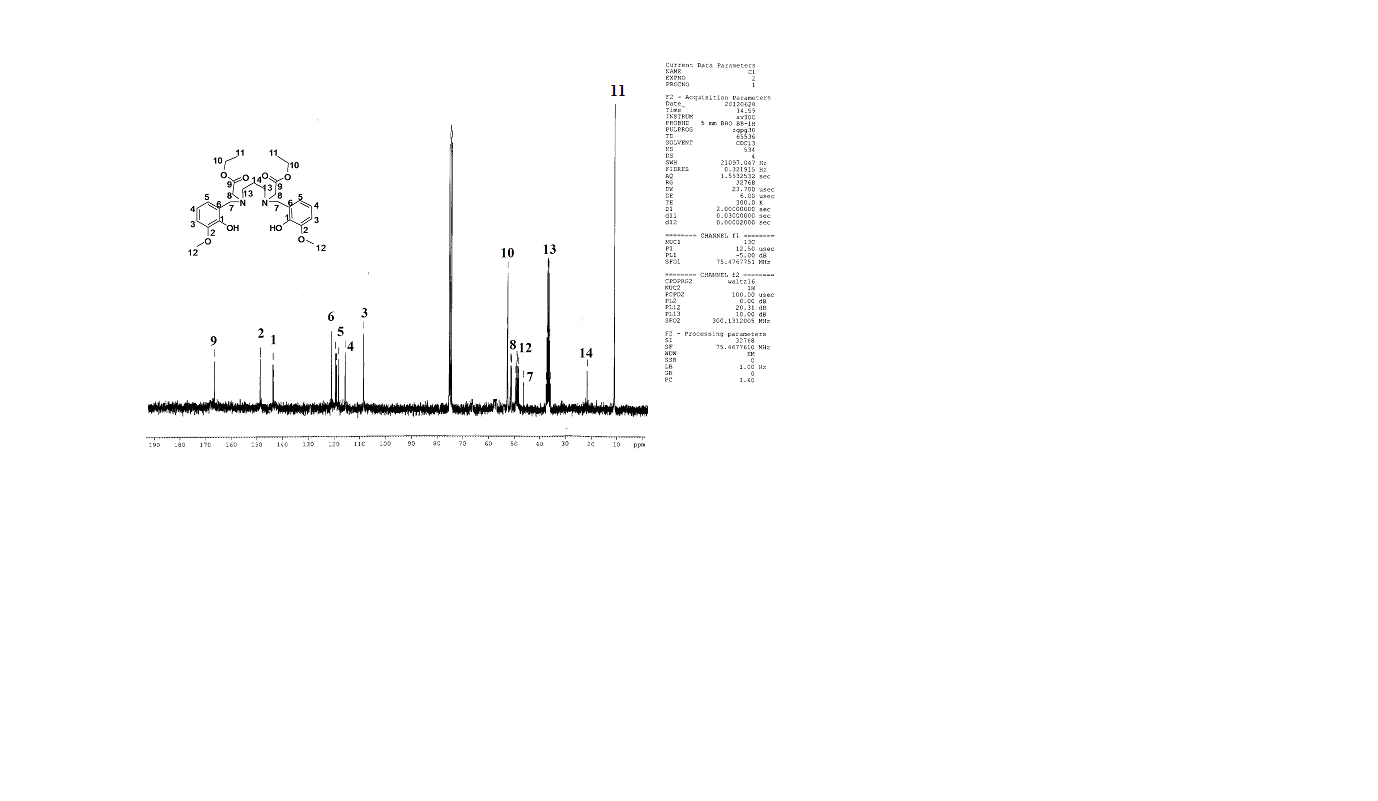
**

**FIGURE S3:** ^13^C NMR spectrum of L in CDCl_3_.


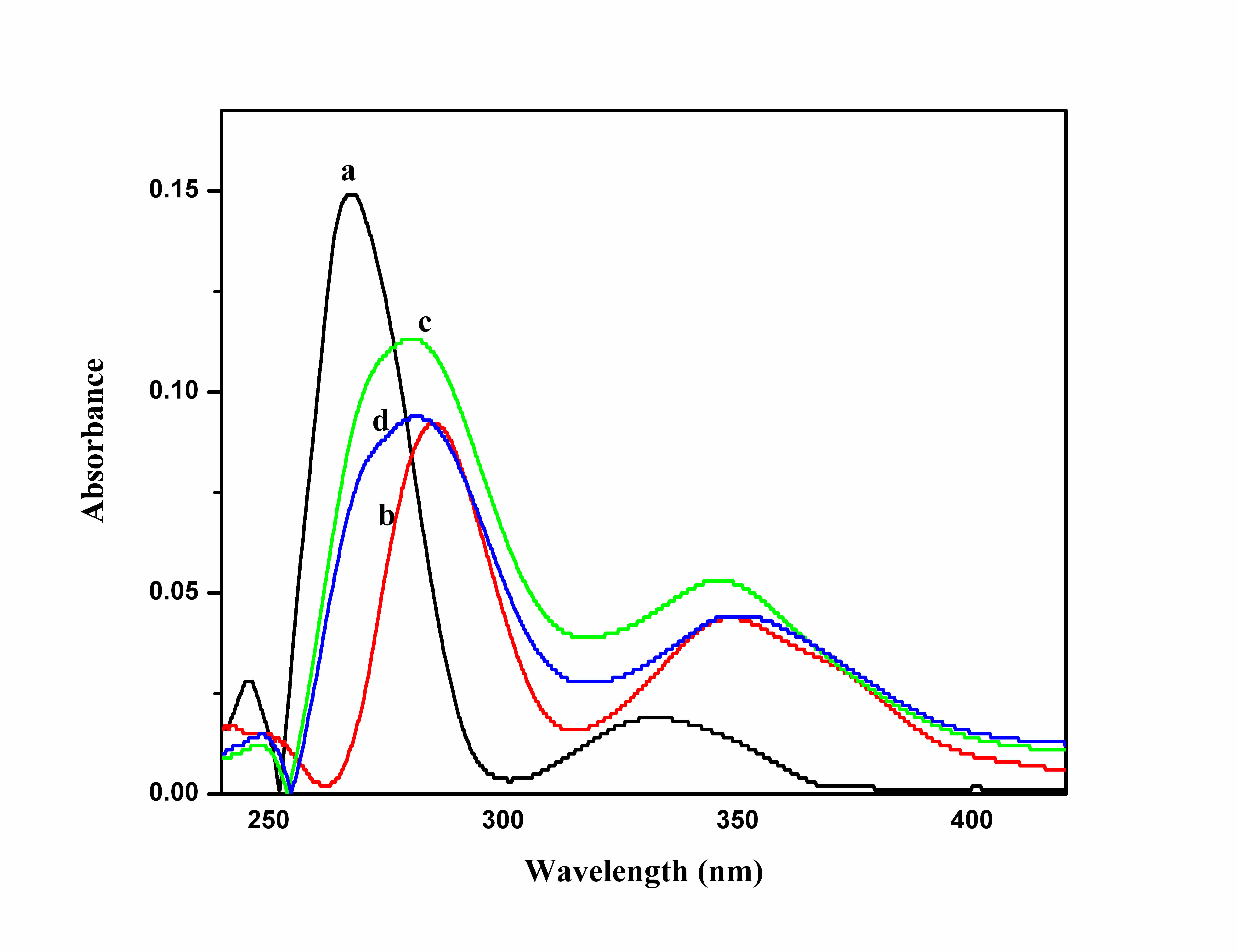


**FIGURE S4:** Electronic spectra of (a) ligand L (b) CuL (c) NiL and (d) ZnL in DMSO.

| 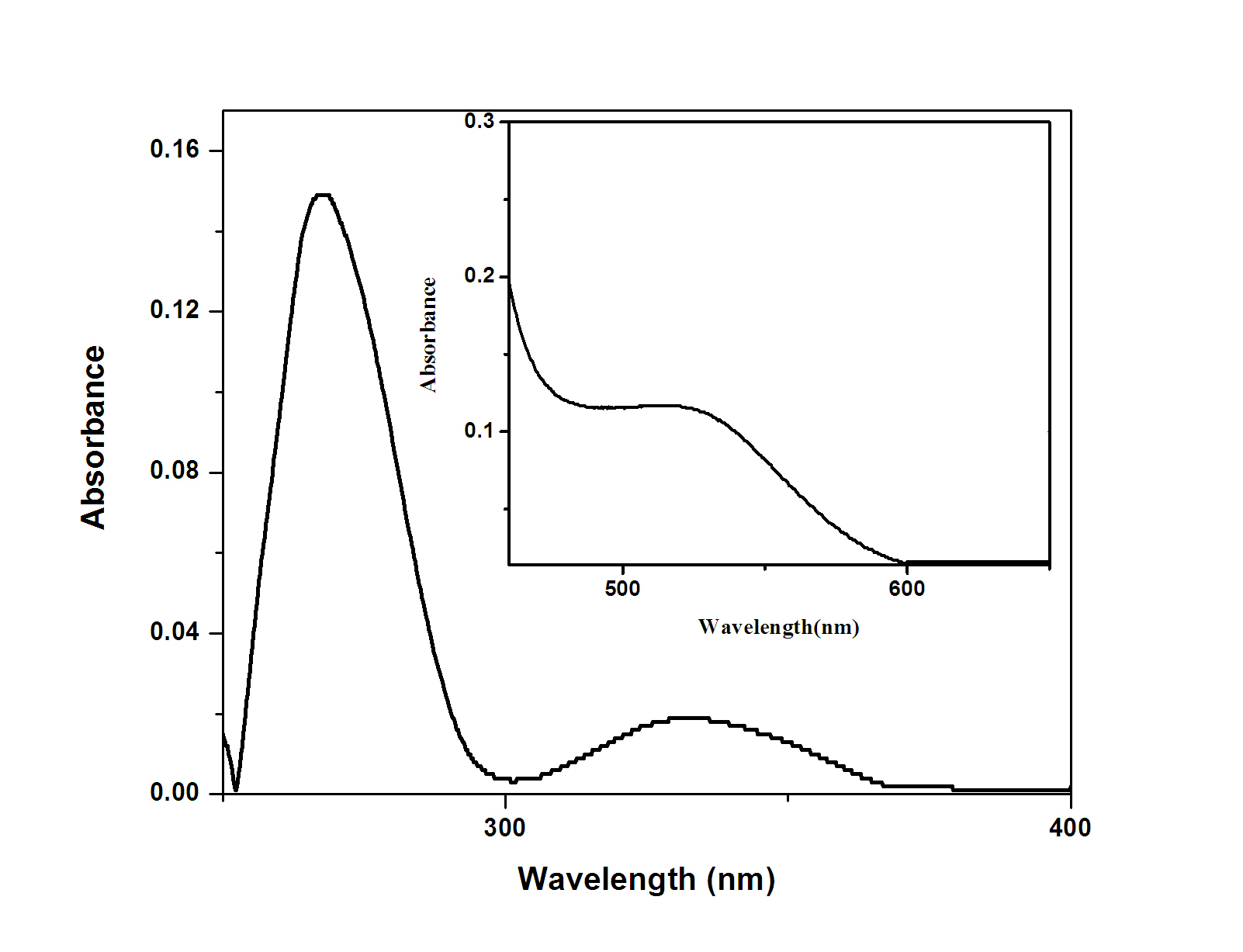 |
| --- |

**FIGURE S5:** UV-Vis spectrum of L in DMSO. Inset shows the d-d band of CuL.


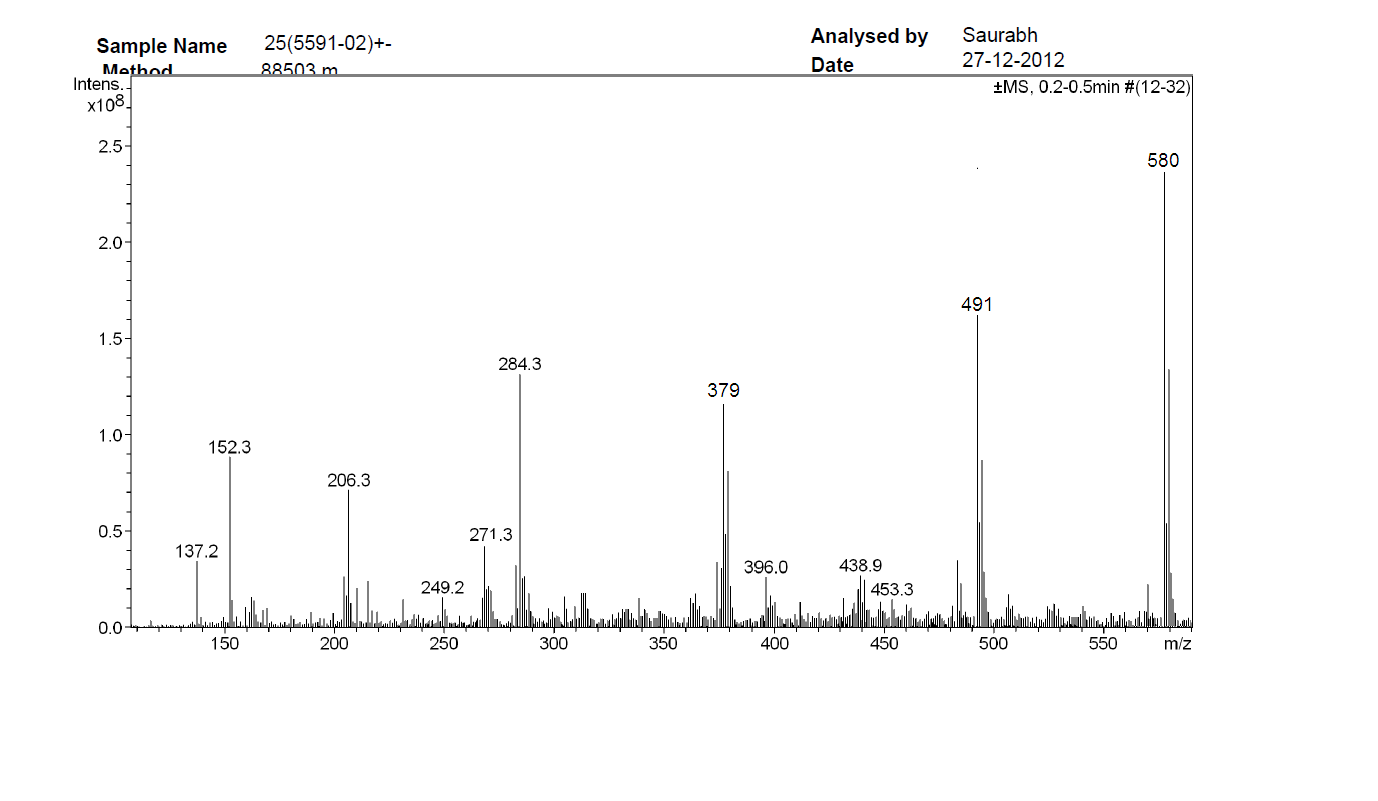


**FIGURE S6:** ESI mass spectrum of CuL.

| 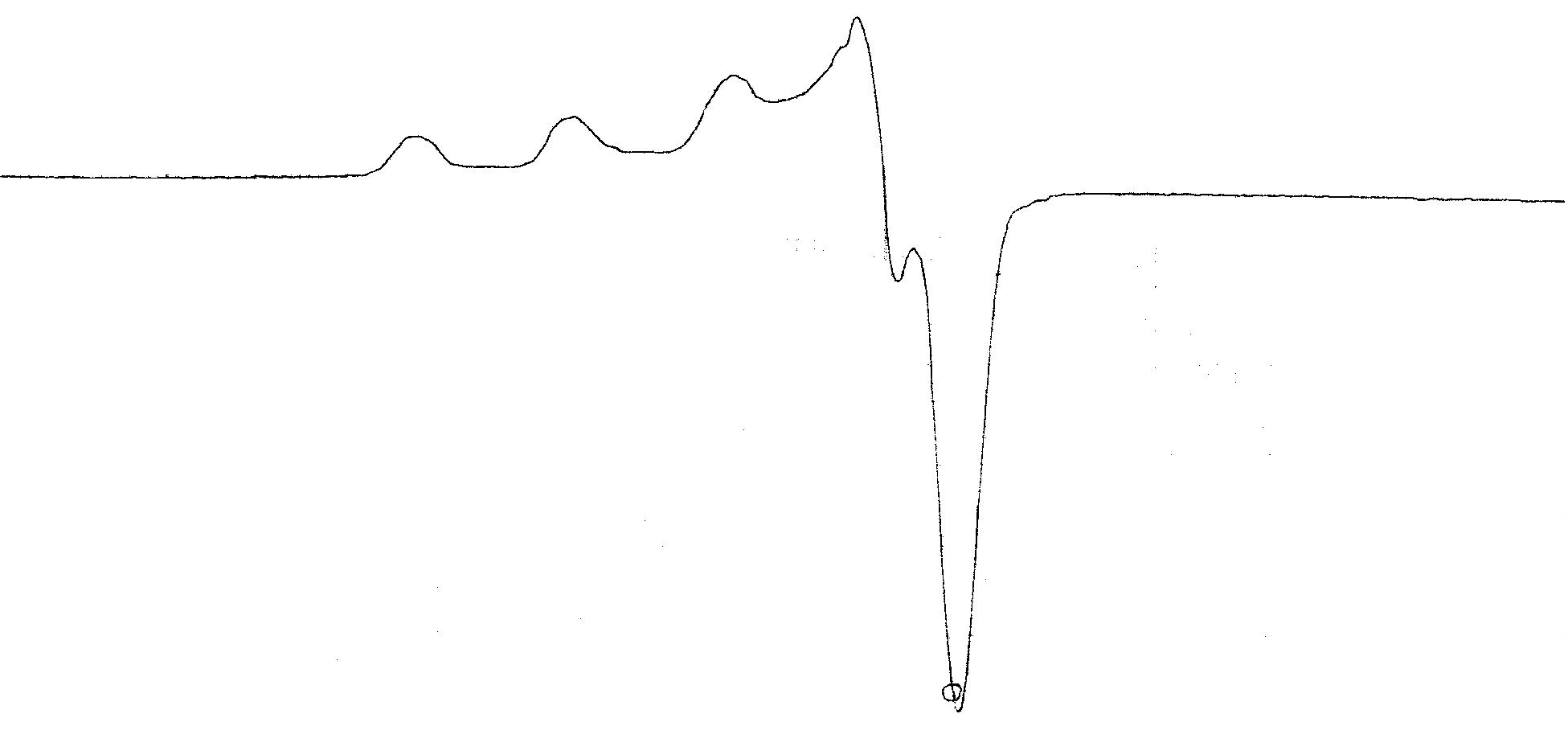 |
| --- |

**FIGURE S7:** EPR spectrum of CuL at liquid nitrogen temperature.

| 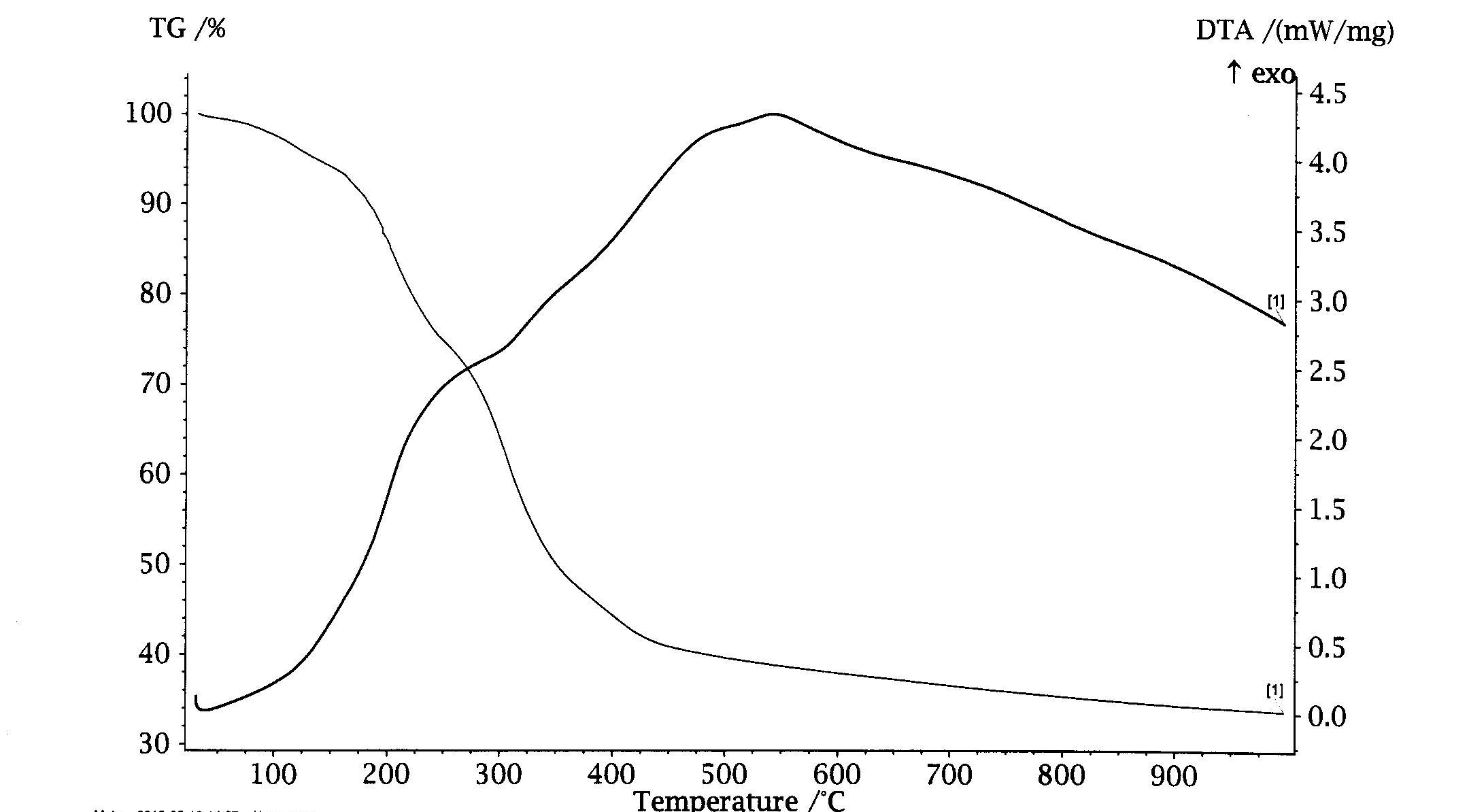  **(a)** |
| --- |
| 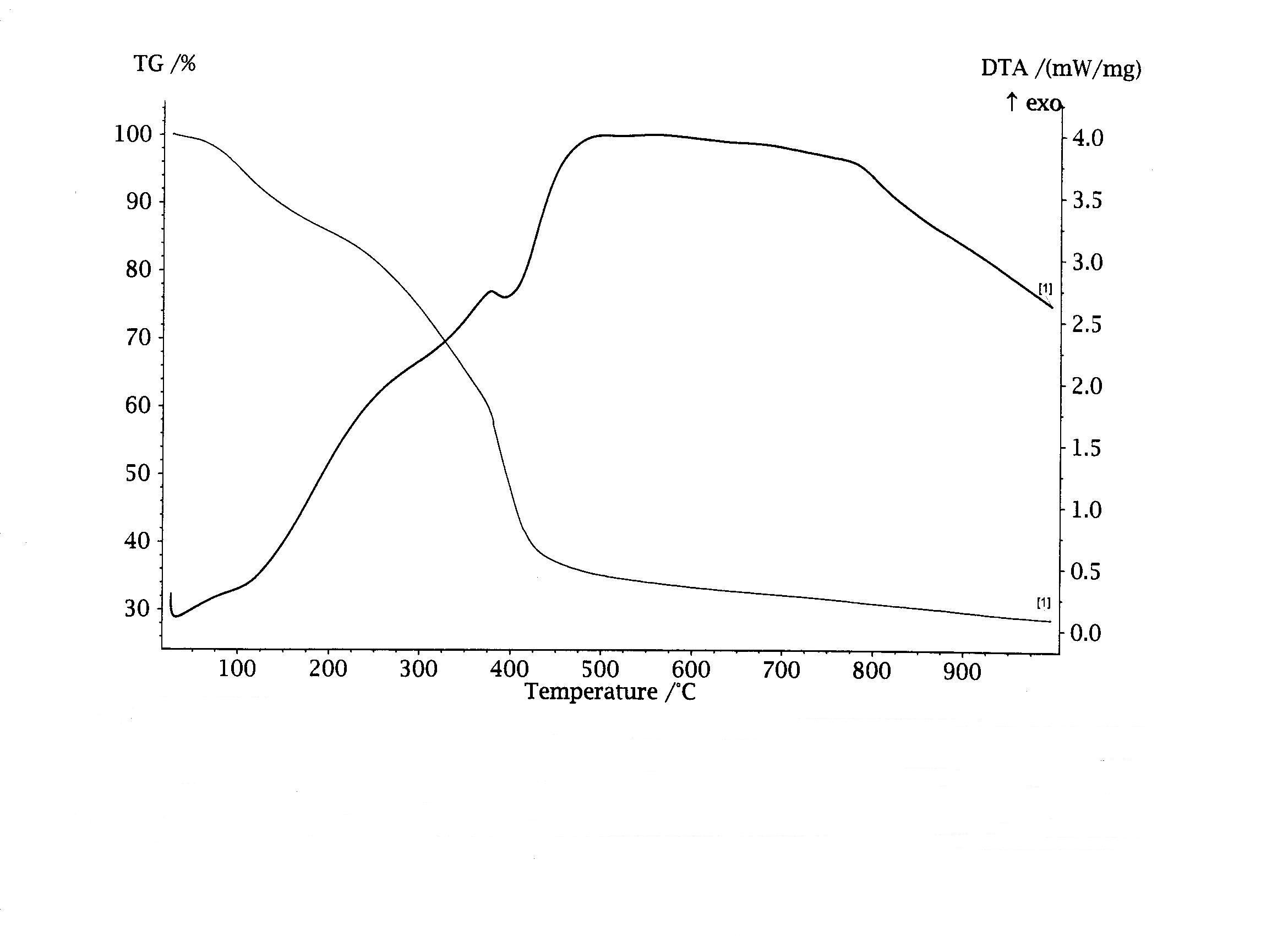  **(b)** |

**FIGURE S8:** Thermogravimetric analysis of (a) CuL and (b) NiL.


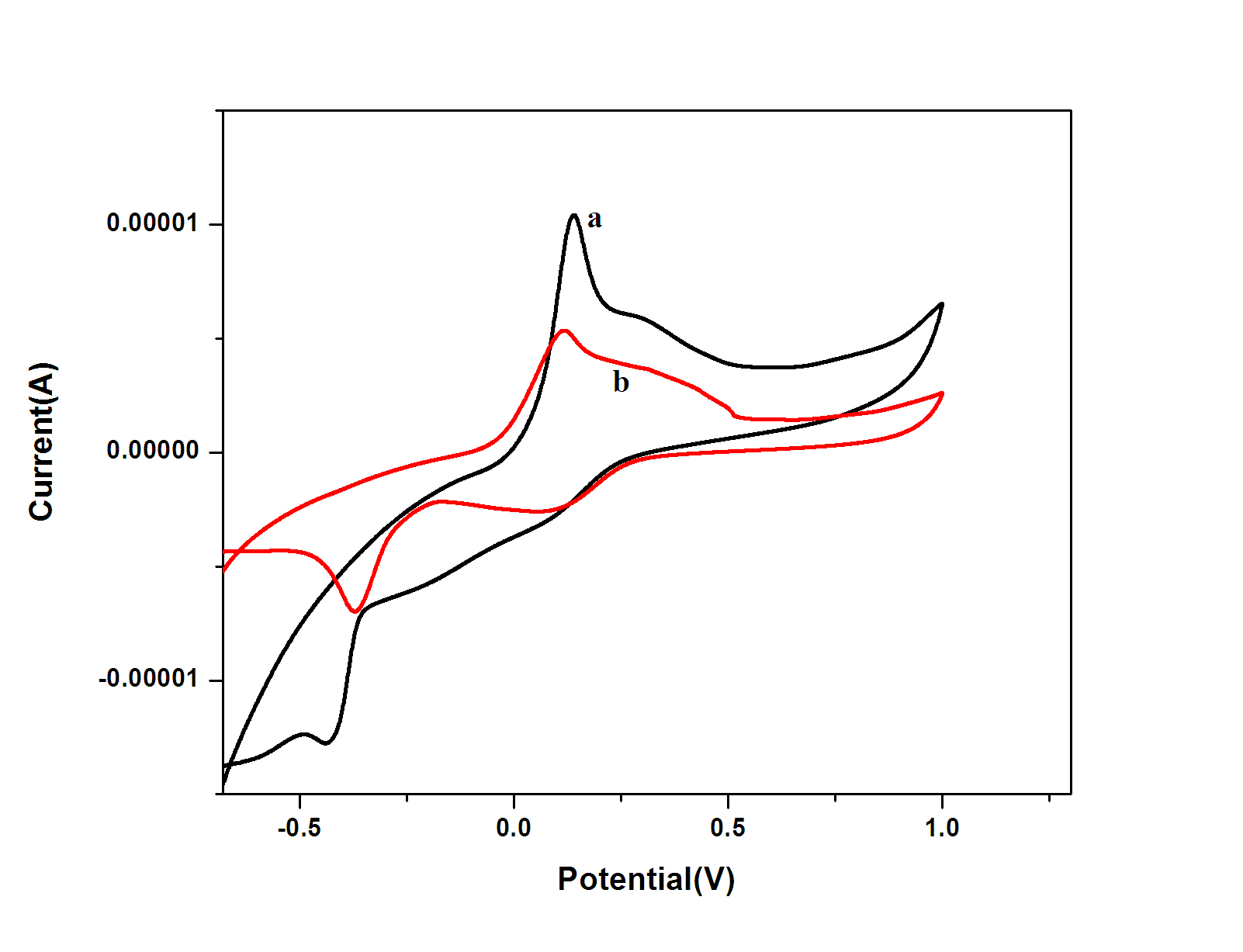


**FIGURE S9:** Redox behaviour of CuL both in the (a) absence and (b) presence of CT DNA using cyclic voltammogram. Supporting electrolyte 5 mM Tris HCl, 50 mM NaCl, pH=7.1. Scan rate 100 mVs^-1^.


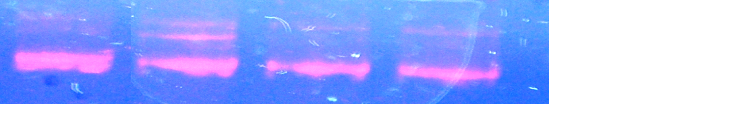


**I**

**III**

**II**

**Lane 1 2 3 4**

**FIGURE S10:** Gel diagram showing the cleavage of pUC 19 DNA by the metal complexes. Lane 1: DNA+H_2_O_2_ (control); Lane 2: DNA+CuL+H_2_O_2_; Lane 3:DNA+NiL+H_2_O_2_; Lane 4: DNA+ZnL+ H_2_O_2._.


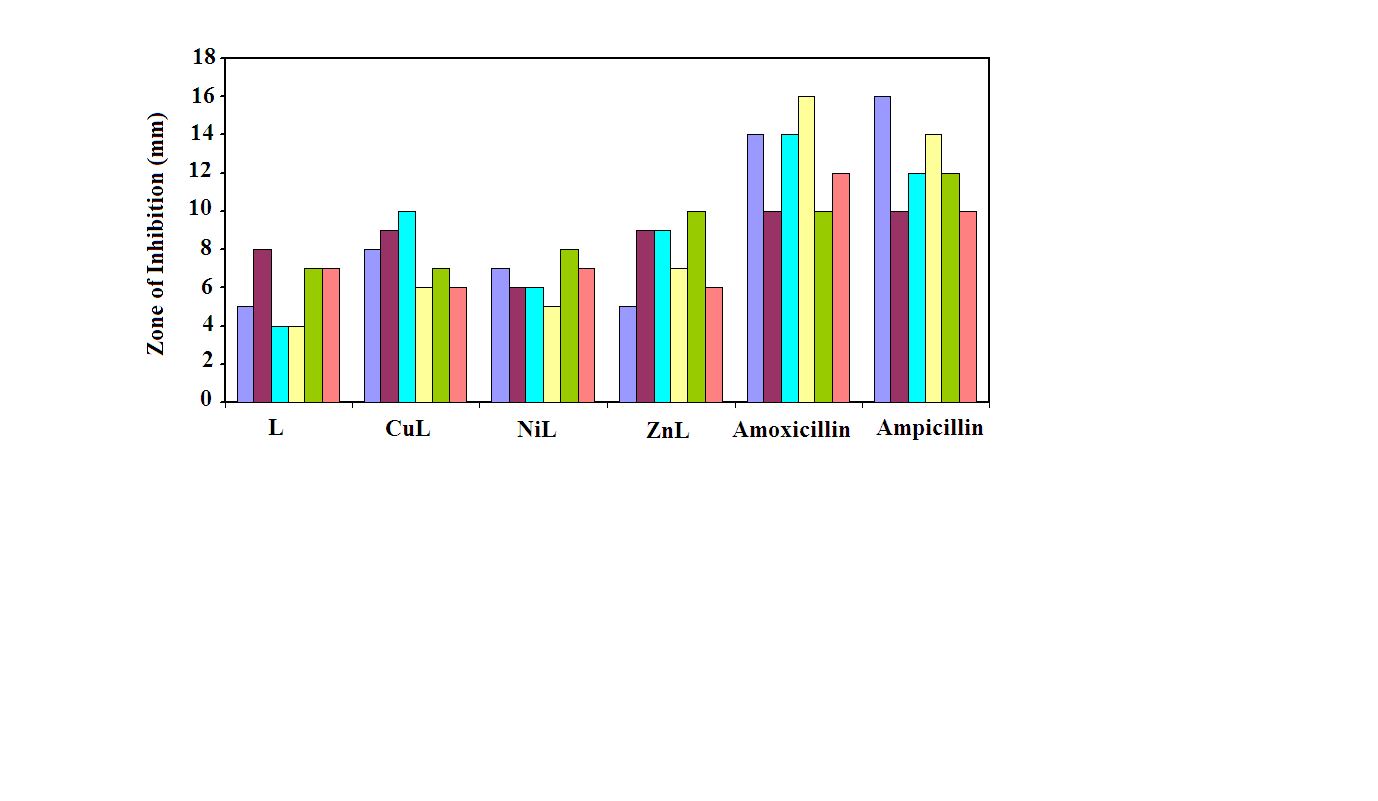


**FIGURE S11:** Antibacterial activities of ligand and their metal complexes by well diffusion method*.*

**
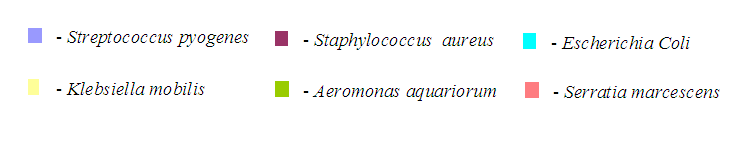
**

**TABLE S1:** ^1^H NMR spectral data of ligand (L) and its NiL and ZnL complexes.

| Numbering | L (δ in ppm) | NiL (δ in ppm) | ZnL (δ in ppm) |
| --- | --- | --- | --- |
| H2,H3,H4 | 6.58-6.87 | 6.57-6.94 | 6.55-6.91 |
| H5 | 3.86 | 4.37 | 4.35 |
| H6 | 2.63 | 3.20 | 3.16 |
| H7 | 1.77 | 1.85 | 1.79 |
| H8 | 3.35 | 3.70 | 3.72 |
| H9 | 4.16 | 4.21 | 4.19 |
| H10 | 1.43 | 1.51 | 1.51 |
| H11 | 3.86 | 3.91 | 3.92 |

**TABLE S2:** ^13^C NMR spectral data of ligand (L) and its Zn complex (ZnL).

| Numbering | L (δ in ppm) | ZnL (δ in ppm) |
| --- | --- | --- |
| C1 | 156.51 | 167.21 |
| C2 | 151.41 | 151.55 |
| C3 | 116.21 | 117.95 |
| C4 | 123.40 | 122.71 |
| C5 | 125.89 | 124.41 |
| C6 | 127.17 | 130.79 |
| C7 | 54.20 | 51.09 |
| C8 | 59.12 | 60.31 |
| C9 | 174.12 | 172.19 |
| C10 | 61.85 | 61.58 |
| C11 | 13.95 | 14.12 |
| C12 | 56.81 | 56.49 |
| C13 | 45.37 | 41.18 |
| C14 | 29.30 | 29.62 |

**TABLE S3:** Cyclic voltammetric data of copper complex in DMSO containing TBAP.

| Compound | Scan rate | Ep_a_ (V) | Ep_c_ (V) | ∆Ep (V) | Ip_a_/Ip_c_ |
| --- | --- | --- | --- | --- | --- |
| CuL | 25 | 0.552 | 0.250 | 0.302 | 1.1 |
|  | 50 | 0.575 | 0.268 | 0.307 | 1.4 |
|  | 75 | 0.585 | 0.262 | 0.323 | 1.3 |
|  | 100 | 0.604 | 0.260 | 0.344 | 1.1 |
|  | 125 | 0.608 | 0.258 | 0.350 | 1.3 |

**TABLE S4:** Electrochemical parameters for the interaction of CT DNA with Cu(II) complex.

| Compound | Ep_c_ (V) | | Ep_a_(V) | | E_1/2_(V) | | ΔE_p_(V) | | Ip_a_/Ip_c_ | |
| --- | --- | --- | --- | --- | --- | --- | --- | --- | --- | --- |
|  | Free | Bound | Free | Bound | Free | Bound | Free | Bound | Free | Bound |
| CuL | -0.438 | -0.364 | 0.137 | 0.118 | -0.151 | -0.123 | 0.575 | 0.482 | 0.90 | 0.46 |
